# Genetic Variants in miRNAs Are Associated With Risk of Non-syndromic Tooth Agenesis

**DOI:** 10.3389/fphys.2020.01052

**Published:** 2020-08-21

**Authors:** Min Gu, Xin Yu, Liwen Fan, Guirong Zhu, Fan Yang, Shu Lou, Lan Ma, Yongchu Pan, Lin Wang

**Affiliations:** ^1^Jiangsu Key Laboratory of Oral Diseases, Nanjing Medical University, Nanjing, China; ^2^Department of Orthodontics, Affiliated Stomatological Hospital, Nanjing Medical University, Nanjing, China; ^3^Department of Dentistry, The Third Affiliated Hospital of Soochow University, The First People’s Hospital of Changzhou, Changzhou, China; ^4^State Key Laboratory of Reproductive Medicine, Nanjing Medical University, Nanjing, China

**Keywords:** miRNA, single nucleotide polymorphism, tooth agenesis, *miR-605-5p*, *MDM2*, *p53*

## Abstract

Non-syndromic tooth agenesis (NSTA) is one of the most common dental abnormalities. MiRNAs participated in the craniofacial and tooth development. Therefore, single nucleotide polymorphisms (SNPs) in miRNA genes may contribute to the susceptibility of non-syndromic tooth agenesis. Here, a total of 625 non-syndromic tooth agenesis cases and 1,144 healthy controls were recruited, and four miRNA SNPs (*miR-146a*/rs2910164, *miR-196a2*/rs11614913, *pre-miR-605*/rs2043556, *pre-miR-618*/rs2682818) were genotyped by the TaqMan platform. Rs2043556 showed nominal associations with risk of non-syndromic tooth agenesis (*P*_Add_ = 0.021) in the overall analysis, as well as upper lateral incisor agenesis (*P*_Add_ = 0.047) and lower incisor agenesis (*P*_Add_ = 0.049) in the subgroup analysis. Notably, its significant association with upper canine agenesis was observed (*P*_Add_ = 0.0016). Rs2043556 affected the mature of *miR-605-3p* and *miR-605-5p* while dual-luciferase report analysis indicated that *MDM2* was the binding target of *miR-605-5p.* Our study indicated that *pre-miR-605* rs2043556 was associated with risk of non-syndromic tooth agenesis.

## Introduction

Tooth agenesis (TA) is one of the most common developmental abnormalities in humans, defined as the absence of one or more permanent teeth, which affects the esthetic, masticatory and occlusal functions of humans. Congenital tooth deficiency affects approximately 20% of the population worldwide (Vastardis, 2000). TA can occur as a syndromic form, which is associated with other genetic diseases, such as non-lethal Raine syndrome (Acevedo et al., 2015), while the more common type is non-syndromic tooth agenesis (NSTA), which occurs as an isolated condition without other birth defects (Fauzi et al., 2018).

Most of human tooth agenesis cases are caused by genetic factors and mutations in *AXIN2*, *EDA*, *LRP6*, *MSX1*, *PAX9*, *WNT10A*, *WNT10B*, and *BMP4* are known to cause tooth agenesis (Yu et al., 2016, 2019a,b; Wong et al., 2018). Single nucleotide polymorphisms (SNPs) are a type of DNA sequence polymorphism caused by variation in a single nucleotide at the genome level (Botstein and Risch, 2003). Previous studies indicated that genomic SNPs were associated with tooth agenesis. For instance, (Liu et al., 2013) found an association between rs929387 of *GLI3* and non-syndromic tooth agenesis in Chinese Han individuals. In addition, our previous study indicated that rs17563 (Gong et al., 2015), rs15705 and rs317250 (Lu et al., 2016) of *BMP4* were also associated with non-syndromic tooth agenesis.

miRNA, a variety of small non-protein-coding RNAs with a length of approximately 22 nucleotides encoded by endogenous genes, provides an efficient pathway for the regulation of gene expression at the post-transcriptional level (Sehic et al., 2017). In the past few years, the relationship between miRNA and human diseases had been extensively studied. The influence of miRNA on tooth growth and development, as well as tooth-related diseases, had also attracted the wide attention of oral biologists. Studies showed that the *miR-200* family was involved in the regulation of epithelial stem cells and that *miR-200c* regulated tooth enamel formation by influencing signal transduction among ameloblasts (Peng et al., 2012). *MiR-21* was also associated with the osteogenesis of alveolar bone (Liu et al., 2014). In addition, *miR-34a* interacted with notch signaling and promoted both odontogenic and osteogenic differentiation of apical papilla stem cells (SCAPs) (Sun et al., 2014). These studies collectively showed the important role of miRNA in tooth growth and development.

Single nucleotide polymorphisms in miRNA genes may alter pre-miRNA processing or maturation and affect their target specificity (Ha and Kim, 2014), thus resulting in phenotypic differences. Specifically, SNPs in pre-miRNA or pre-miRNA gene regions may affect the expression of mature miRNA, while SNPs around mature miRNA or regions that target genes bind to may affect the binding of miRNA-mRNA (Kroliczewski et al., 2018). Any of the above situations may interfere with the interaction of miRNA-target genes, thus affecting the occurrence of diseases. For instance, (Ghanbari et al., 2016) identified associations of rs897984 in *miR-4519* and rs11651671 in *miR-548at-5p* with Parkinson’s disease. In addition, rs11614913 in *miR-196a2*, rs2910164 in *miR-146a*, and rs3746444 in *miR-499* contributed to the risk of prostate cancer in Asian descendants (Mi et al., 2018). However, to the best of our knowledge, the associations between miRNA gene SNPs and TA susceptibility have not been explored.

With the aim of addressing this issue, here, we selected four miRNA gene SNPs to evaluate their associations with the risk of non-syndromic tooth agenesis in a case-control study and explored the potential underlying mechanism.

## Materials and Methods

### Human Subjects

This study consisted of 625 non-syndromic tooth agenesis cases (The average number of missing teeth per person is 1.62, including 6 oligodontia cases and 619 hypodontia cases. Tooth missing pattern was shown in Supplementary Table S1) and 1,144 healthy controls from the Affiliated Stomatological Hospital of Nanjing Medical University between October 2005 and June 2017. All of the participants were from Nanjing and surrounding cities in south-eastern China.

Informed consent to participate in this study was obtained from all enrolled subjects. The controls and cases were identified by two dentists according to panoramic radiographs, dental examinations, and treatment records. The inclusion criteria of healthy controls were (1) full permanent dentition from the third molar to the contralateral third molar and (2) no metal or gold crown restorations. The non-syndromic tooth agenesis cases had at least one missing tooth, excluding the third molar. Any subjects with additional non-dental abnormalities, such as cleft lip and/or cleft palate (CL/P) or other syndromes, and missing teeth caused by acquired reasons, such as trauma, extraction or orthodontic treatment, were excluded from our study. We collected 2 ml of venous blood from all participating individuals using an anticoagulant vacutainer tube with EDTA. This study was approved by the Nanjing Medical University Institution Review Board (PJ2004-030-001), and all methods and procedures followed the Declaration of Helsinki (version 2002) and additional requirements.

### SNP Selection

Based on our previous study (Pan et al., 2018), we selected four miRNA SNPs (*miR-146a*/rs2910164, *miR-196a2*/rs11614913, *pre-miR-605*/rs2043556, and *pre-miR-618*/rs2682818) of them that were identified from miRBase (version10.0)^1^, dbSNP^2^, HapMap^3^, and Patrocles^4^, that met the following two criteria: (a) SNPs located in the pre-miRNA or mature sequences, (b) the difference in Gibbs binding free energy between the two alleles (ΔΔG) ≥ 2.60 KJ/mol, as the energy parameter ΔΔG may affect the mature miRNA products (Gong et al., 2012) and (c) minor allele frequency (MAF) ≥5% in Chinese population. *Pre-miR-923*/rs47960429 in our previous study was excluded in this study because *miR-923* was a fragment of 28S rRNA in the latest version of miRbase (vision 22.0). Therefore, we chose four SNPs for genotyping (Supplementary Table S2).

### DNA Extraction and Genotyping

DNA extraction was conducted according to the protocol of the TIANamp Genomic DNA Kit (TIANGEN, Beijing) (Fan et al., 2018). DNA samples were stored at −80°C for further manipulation after purity and concentration were measured.

Single nucleotide polymorphisms were genotyped by polymerase chain reaction with the TaqMan-MGB method performed using the ABI-Prism 7900HT Sequence Detection System (Applied Biosystems, Foster City, CA, United States). The reaction system includes a pair of upstream and downstream primers and two probes (Supplementary Table S3). Genotyping results were analyzed by SDS software (version 2.4.1) and reviewed by two independent investigators in a blind manner. Samples that failed to be genotyped were removed. 10% samples were randomly selected for confirmation, and the results were 100% concordant.

### Predicting the Potential Binding Targets of miRNAs

To further find the target gene of *miR-605*, prediction was made by four databases (TargetScan, miRDB, miRTarBase, and miRWalk) according to the following criteria: (1) consistently predicted by all four databases and (2) reported to be potentially associated with tooth development.

### Cell Culture

The HEK-293 (human embryonic kidney 293 cells) and COS-7 cells (Africa green monkey kidney cells) were cultured, respectively, in Eagle’s minimum essential medium (EMEM) and Dulbecco’s modified eagle’s medium (DMEM) comprised of 10% fetal bovine serum (FBS), 100 U/ml penicillin and 100 mg/ml of streptomycin at 37°C in 5% CO_2_. The culture medium was replaced every other day.

### Transfection and Quantitative Real-Time PCR

HEK-293 cells and COS-7 cells, seeded into 12-well culture plates, were instantly transfected with vector DNA containing either the rs2043556 A or G allele and *miR-605-5p* mimics by Lipofectamine 2000 (Invitrogen, Carlsbad, CA, United States). After 48 h, the cells were collected for RNA extraction using RNA extraction kit (Takara, Shiga, Japan) and reverse transcribed into single-stranded cDNA by cDNA synthesis kit (Takara, Shiga, Japan). U6 snRNA was used as corresponding internal controls for miRNA and GAPDH was for gene. Real-time quantitative PCR was performed using Power SYBR Green on a 7900 Real-Time PCR System (Applied Biosystems, Foster City, CA, United States). Data were collected and analyzed by the 2^–ΔΔCt^ method for qualification of the relative expression levels of mature *miR-605* and *MDM2.*

### Construction of Reporter Plasmids and Dual-Luciferase Reporter Assay

The *MDM2* 3′-UTR wild type fragment (407 bp) was inserted at the Nhel-Xhol restriction site downstream of the luciferase gene in the pmirGLO vector (Supplementary Figure S1). HEK-293 and COS-7 cells, seeded into 24-well culture plates, were transfected with the reporter plasmids containing the *MDM2* 3′-UTR fragment and *miR-605-5p* mimic using Lipofectamine 2000 (Invitrogen, Carlsbad, CA, United States). The luciferase activity in the lysates was quantified with a dual-luciferase reporter assay system after transfection for 24 h (Promega, Madison, WI, United States). The ratio of firefly luciferase to renilla luciferase activity was evaluated, representing the binding activity of miRNA to a given 3′-UTR.

### Western Blot Analysis

The cells were lysed by RIPA buffer (Beyotime) and the concentration of protein was measured using BCA Kit (Beyotime). We separated the equivalent amount of protein (20 μg protein/lane) on 8% SDS-PAGE and transfer them to PVDF membrane (Millipore). The membranes were blocked with 5% non-fat milk for 2 h at room temperature and then incubated at 4°C overnight with primary antibodies including anti-MDM2 (Santa Cruz, sc-965, 1:800) and anti-GAPDH (Beyotime, AG019, 1:1000). After incubation with horseradish peroxidase-conjugated secondary antibodies (1:10000), the protein bands were visualized by chemiluminescence reagents (Merck Millipore, WBKLS0050).

### *In silico* Analysis

We retrieved and downloaded three datasets, including GSE138180, GSE42589, and GSE48150 from the GEO database^5^, a public database of microarray and sequencing data that contained a large number of disease-related gene expressions. GSE138180 explored the roles of miRNAs in odontogenic differentiation of human dental pulp stem cells (DPSCs). GSE42589 showed gene expression data on dental pulp stem cells from 13 samples while GSE48150 reported gene expression of tooth germs at the cap stage from 12-week-old human embryonic oral cavity.

### Statistical Analysis

Data were subsequently processed and analyzed with PLINK software (version 1.07). The chi-square test and Student’s *t* test were used to analyze the age and gender distribution between the case and control group. Odds ratio (OR) and confidence intervals (CIs) calculated by logistic regression analysis were used to determine whether any SNPs were preferentially associated with the risk of non-syndromic tooth agenesis under additive and allelic models. Hardy-Weinberg equilibrium (HWE) was evaluated in the controls by a goodness-of-fit χ^2^ test. *P* value ≤ 0.05 was designated as nominal association while *P* value ≤ 0.0025 (0.05/4/5) after the Bonferroni correction was appointed as significant association. Luciferase activity analysis and qRT-PCR results were calculated by Student’s *t* test and one-way analysis of variance (ANOVA).

## Results

### Characteristics of the Samples

A total of 625 cases (205 males and 420 females, mean age: 15.91 ± 8.34) and 1,144 controls (415 males and 729 females, mean age: 15.88 ± 7.48) were recruited, and their information is shown in Supplementary Table S1. There was no significant difference in gender distribution (*P* = 0.143) between the case and control groups.

The distribution of missing teeth in the maxillary (*N* = 219, 30.4%) and mandibular regions (*N* = 501, 69.6%) is shown in Supplementary Table S1. Among the samples we collected, mandibular incisors were the most common missing teeth (*N* = 362, 50.3%), followed by mandibular premolars (*N* = 139, 19.3%), maxillary lateral incisors (*N* = 96, 13.3%), maxillary premolar (*N* = 72, 10.0%) and maxillary canine (*N* = 51, 7.1%).

### Overall Analysis Between SNPs and NSTA Risk

A total of 614 cases and 1,112 healthy controls were successfully genotyped in the present study. The genotype and allele distributions of each SNP among non-syndromic tooth agenesis cases and controls were calculated. The observed genotype frequencies in the control group were consistent with HWE (*P* > 0.01). We evaluated the association between non-syndromic tooth agenesis risk and SNPs under additive and allelic models.

As shown in Table 1, nominal associations were detected between rs2043556 and non-syndromic tooth agenesis risk in the overall analysis under additive (*P* = 0.021, OR = 1.20, 95% CI = 1.03–1.40) and allelic (*P* = 0.021, OR = 1.20, 95% CI = 1.03–1.40) models. None of any association was observed for the other three SNPs (*P*_Add_ = 0.776 for rs2910164, *P*_Add_ = 0.557 for rs11614913, *P*_Add_ = 0.553 for rs2682818).

**TABLE 1:** Overall associations between the four SNPs and non-syndromic tooth agenesis (NSTA) susceptibility.

SNP	Genotype	Controls *N* = 1112	NSTA cases *N* = 614	Models	*P*	OR (95% CI)
rs2910164(G > C)	GG/GC/CC	368/577/167	213/293/108	Additive	0.776	1.02 (0.88–1.18)
	G/C	1313/911	719/509	Allele	0.781	1.02 (0.89–1.18)
rs2043556(A > G)	AA/AG/GG	600/436/76	304/250/60	Additive	**0.021**	**1.20 (1.03–1.40)**
	A/G	1636/588	858/370	Allele	**0.021**	**1.20 (1.03–1.40)**
rs11614913(T > C)	TT/TC/CC	312/572/228	184/305/125	Additive	0.557	0.96 (0.83–1.10)
	T/C	1196/1028	673/555	Allele	0.562	0.96 (0.83–1.10)
rs2682818(C > A)	CC/CA/AA	614/426/72	328/246/40	Additive	0.553	1.05 (0.89–1.23)
	C/A	1654/570	902/326	Allele	0.556	1.05 (0.90–1.23)

*SNP, single nucleotide polymorphism; NSTA, non-syndromic tooth agenesis; OR, odds ratio; CI, confidence interval; Bold values, significant values.*

### Subgroup Analysis With the Position of Missing Teeth

All cases were divided into five subgroups according to different positions of missing teeth: upper lateral incisor agenesis (*N* = 95, 13.4%), upper canine agenesis (*N* = 50, 7.1%), upper premolar agenesis (*N* = 72, 10.2%), lower incisor agenesis (*N* = 354, 49.9%) and lower premolar agenesis (*N* = 138, 19.5%). Taking upper lateral incisor agenesis for example, it refers to the subjects with congenitally missing upper lateral incisor with or without other types of missing teeth.

As shown in Table 2, rs2043556 was nominally associated with upper lateral incisor agenesis (*P*_Add_ = 0.047, OR = 1.38, 95% CI = 1.01–1.89; *P*_*Allelic*_ = 0.046, OR = 1.38, 95% CI = 1.01–1.89) and lower incisor agenesis (*P*_Add_ = 0.049, OR = 1.21, 95% CI = 1.00–1.4; *P*_*Allelic*_ = 0.049, OR = 1.21, 95% CI = 1.00–1.45). Furthermore, its significant association with upper canine agenesis was detected (*P*_Add_ = 0.0016, OR = 1.95, 95% CI = 1.29–2.95; *P*_*Allelic*_ = 0.0016, OR = 1.93, 95% CI = 1.28–2.91). However, the subgroup analysis did not reveal any significant associations between rs2910164, rs11614913 and rs2682818 and susceptibility of non-syndromic tooth agenesis.

**TABLE 2:** Association of the four miRNA gene SNPs with the risk of different types of teeth agenesis.

**SNP**	**Models**	**Controls *N* = 1112**	**Upper lateral incisor agenesis *N* = 95**	**Upper canine agenesis *N* = 50**	**Upper premolar agenesis *N* = 72**	**Lower incisor agenesis *N* = 354**	**Lower premolar agenesis *N* = 138**
rs2910164	GG/GC/CC	368/577/167	29/46/20	19/21/10	23/35/14	116/172/66	58/59/21
	Additive GG/GC/CC	*P* OR (95% CI)	0.233 1.21 (0.89–1.64)	0.994 1.00 (0.66–1.53)	0.495 1.13 (0.79–1.61)	0.340 1.09 (0.91–1.30)	0.152 0.82 (0.63–1.07)
	Allele G/C	*P* OR (95% CI)	0.248 1.19 (0.88–1.61)	0.994 1.00 (0.67–1.51)	0.510 1.12 (0.80–1.57)	0.353 1.08 (0.91–1.29)	0.164 0.83 (0.64–1.08)
rs2043556	AA/AG/GG	600/436/76	45/37/13	17/25/8	39/31/2	173/148/33	78/47/13
	Additive AA/AG/GG	*P* OR (95% CI)	**0.047** **1.38 (1.01–1.89)**	**0.0016** **1.95 (1.29–2.95)**	0.570 0.89 (0.60–1.32)	**0.049** **1.21 (1.00–1.45)**	0.997 1.00 (0.75–1.33)
	Allele A/G	*P* OR (95% CI)	**0.046** **1.38 (1.01–1.89)**	**0.0016** **1.93 (1.28–2.91)**	0.573 0.89 (0.60–1.32)	**0.049** **1.21 (1.00–1.45)**	0.997 1.00 (0.75–1.33)
rs11614913	TT/TC/CC	312/572/228	28/45/22	17/21/12	24/31/17	100/181/73	43/71/24
	Additive TT/TC/CC	*P* OR (95% CI)	0.868 1.03 (0.76–1.39)	0.808 0.95 (0.63–1.43)	0.798 0.96 (0.68–1.35)	0.986 1.00 (0.84–1.19)	0.320 0.88 (0.68–1.14)
	Allele T/C	*P* OR (95% CI)	0.870 1.03 (0.76–1.38)	0.810 0.95 (0.64–1.42)	0.800 0.96 (0.68–1.34)	0.986 1.00 (0.84–1.18)	0.329 0.88 (0.69–1.14)
rs2682818	CC/CA/AA	614/426/72	50/38/7	30/18/2	41/24/7	185/146/23	74/52/12
	Additive CC/CA/AA	*P* OR (95% CI)	0.598 1.09 (0.78–1.53)	0.414 0.82 (0.50–1.33)	0.840 1.04 (0.71–1.52)	0.428 1.08 (0.89–1.31)	0.496 1.10 (0.83–1.46)
	Allele C/A	*P* OR (95% CI)	0.599 1.09 (0.78–1.53)	0.416 0.82 (0.51–1.33)	0.840 1.04 (0.71–1.53)	0.431 1.08 (0.89–1.31)	0.495 1.10 (0.83–1.46)

*Bold values, significant values; OR, odds ratio; CI, confidence interval.*

### Rs2043556 Affects the Mature of *miR-605-3p* and *miR-605-5p*

**FIGURE 1:**
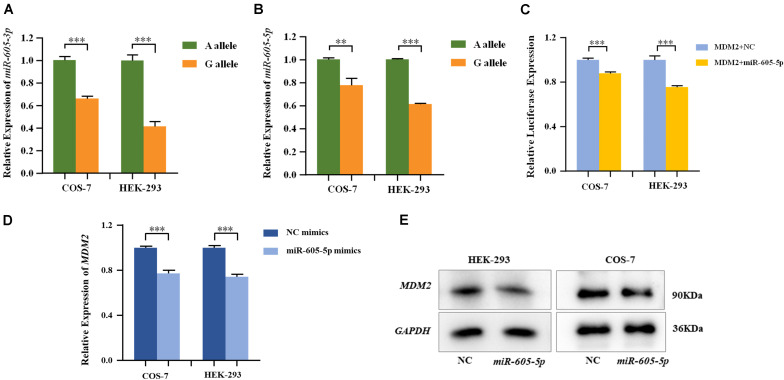
Expression of *miR-605-3p* **(A)** and *miR-605-5p* **(B)** in human cell lines. COS-7 and HEK-293 cells were transfected to express either the *miR-605-3p* A/G allele. The *miR-605-3p* expression levels were estimated by qRT-PCR; *n* = 3 (colonies) for each group and results are shown as mean values with the SD normalized to U6. **(C)** Dual luciferase reporter assays in COS-7 cells and HEK-293 cells demonstrated that *MDM2* was direct target of *miR-605-5p*. *MDM2* mRNA **(D)** and protein **(E)** expression in COS-7 and HEK-293 cells after transfection with miR-605-5p mimics. Transcript levels were analyzed by qPCR and normalized to GAPDH levels (**P < 0.01; ***P < 0.001).

MiRNA plasmids containing pre-miR-605-A and pre-miR-605-G were transiently transfected into HEK-293 and COS-7 cells. The expression levels of mature *miR-605*, including *miR-605-3p* and *miR-605-5p*, were, respectively, tested with qRT-PCR. As shown in Figures 1A,B, the processing efficiency of the pre-miR-605-G plasmid was significantly lower than that of the pre-miR-605-A plasmid in HEK-293 and COS-7 cells (*miR-605-3P: P*_COS–7_ = 8.9E-4, *P*_HEK–293_ = 1.0E-4; *miR-605-5P: P*_COS–7_ = 7.4E-3, *P*_HEK–293_ = 3.6E-7), consistent with the study by Zhou et al. (2019).

### *miR-605-3p* and *miR-605-5p* Expressions in Human Dental Pulp Stem Cells (DPSCs)

GSE138180 contained miRNAs expression of DPSCs in control groups and differentiated groups were cultured with an odontogenic differentiation medium (Supplementary Table S4). Here, we found *miR-605-5p* expression was significantly higher than *miR-605-3p* in both two groups (*P*_control_ = 1.27E-7, *P*_differentiation_ = 1.62E-5). In addition, *miR-605-5p* expression increased after odontogenic differentiation (*P* = 1.12E-3) while *miR-605-3*p showed no significant change (*P* = 3.67E-1). Taken together, *miR-605-5p* played a more important role in tooth development and warranted the further research.

### *MDM2* Was Identified as the Target Gene of *miR-605-5p*

*MDM2*, highly expressed in the odontoblasts in the dental papilla cells of mouse incisors and molars (Zheng et al., 2019), was consistently predicted by four databases to be a favorable target of *miR-605-5p* (Supplementary Figure S2). In addition, *P53*, the canonical substrate of *MDM2*, was expressed in all layers of the human tooth buds (Muica Nagy-Bota et al., 2014). We therefore cloned luciferase reporter plasmids with the *MDM2* 3′-UTR and co-transfected with miRNA in HEK-293 and COS-7 cells. As presented in Figure 1C, luciferase reporter gene analysis showed that the activity of the *MDM2* 3’-UTR reporter gene was significantly decreased by *miR-605-5p* (*P*_COS–7=_6.5E-4, *P*_HEK–293_ = 4.0E-4). In addition, when transfecting *miR-605-5p* mimics into these cell lines, the expression of *MDM2* mRNA and protein were decreased significantly (Figures 1D,E).

### *MDM2* and *P53* Are Expressed During the Cap Stage of Tooth Germs

**FIGURE 2:**
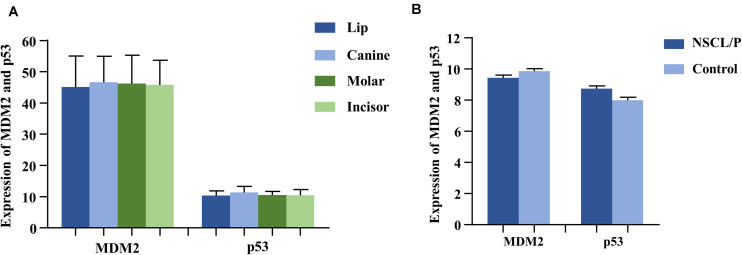
The expression of MDM2 and p53 in human tooth germs **(A)** from GEO datasets (GSE48150) and dental pulp stem cells **(B)** from GEO datasets (GSE42589). NSCL/P, non-syndromic cleft lip with or without cleft palate.

As the gene expression data (GSE48150) in GEO datasets showed, tooth germs of molar, incisor, and canine at the cap stage were, respectively, dissected from 12-week-old human embryonic oral cavity for RNA extraction. We found that both *MDM2* and *P53* were expressed in the dental tissues by multiple transcripts (Figure 2A). Furthermore, *P53* exhibited higher expression in canine germs than in molar (*P* = 0.09) and incisor germs (*P* = 0.04) compared by the paired *t* test (Supplementary Table S5).

### *MDM2 and P53* Expressions in Human Dental Pulp Stem Cells (DPSCs)

Based on the analysis of gene expression data (GSE42589) in DPSCS from GEO datasets, both *MDM2* and *P53* mRNA were detected in DPSCs from 13 samples, including 6 control samples and 7 non-syndromic cleft lip with or without cleft palate (NSCL/P) samples (Figure 2), which often accompanied by tooth agenesis. Interestingly, a significant reverse correlation between *MDM2* and *P53* was observed by Spearman’s correlation (*r* = −0.709, *P* = 0.007).

## Discussion

Single nucleotide polymorphisms in miRNA genes may affect the expression of miRNA and its binding efficiency with target genes, leading to the occurrence of common human diseases (Ryan et al., 2010; Schoen et al., 2017). Existing evidence shows that SNPs in miRNA can be useful markers for a variety of diseases (Hui et al., 2016; Pan et al., 2018). The four SNPs in the present study had been reported in various human diseases. For example, *pre-miR-605/rs2043556* had previously been related to the lung cancer (Xu et al., 2018) and oral squamous cell carcinoma (Miao et al., 2016). *miR-146a/rs2910164* was associated with the non-syndromic orofacial cleft (Pan et al., 2018) and bladder cancer (Wang et al., 2012). *miR-196a2/rs11614913* showed an association with the hepatocellular carcinoma (Zheng et al., 2017) and type 1 diabetes mellitus (Ibrahim et al., 2019). *pre-miR-618*/rs2682818 had been reported in the colorectal cancer (Chen et al., 2018) and lymphomagenesis (Fu et al., 2014). However, their roles in tooth development remain to be explored.

Here, we selected four common variants in miRNA genes and found that rs2043556 was nominally associated with non-syndromic tooth agenesis risk in the overall analysis. The type of non-syndromic tooth agenesis may be associated with specific types of mutation according to the previous study (Williams and Letra, 2018). Jonsson et al. (2018) found two variants, located in *EDA* and *FOXP1*, were associated with agenesis of the maxillary lateral incisors. *EDA* was also identified as a genetic risk factor for maxillary lateral incisor agenesis in another research (Alves-Ferreira et al., 2014). In the present study, rs2043556 showed significant association with the risk of upper canine agenesis and nominal association with the risk of upper lateral incisor and lower incisor agenesis. To the best of our knowledge, this is the first finding of genetic variants contributing to susceptibility of canine agenesis.

Rs2043556 was located in *pre-miR-605* and our study showed that this SNP was associated with differential expression of mature *miR-605* (including *miR-605-3p* and *miR-605-5p*), consistent with the study by Zhou et al. (2017) *Pre-miR-605* had previously been linked to a number of malignancies, such as prostate cancer, breast cancer (Morales et al., 2018), non-small-cell lung cancer (Zhou and Li, 2019), and oral squamous cell carcinoma (Miao et al., 2016), suggesting its potential as a biomarker for cancer. Interestingly, tooth agenesis and cancer development share common molecular pathways and previous study found that almost all types of tumors were more common in families with tooth agenesis (Kuchler et al., 2013).

*MiR-605*, a component in the *P53* gene network, was the first miRNA identified as upregulating *P53* by repressing the translation of *MDM2* (Xiao et al., 2011). One of the core functions of *P53* was to activate apoptosis (Fridman and Lowe, 2003), which existed in all stages of tooth development and played an important role in the development and formation of the final form of the tooth crown. Matalova et al. (2004) found strong expressions of *P53* in the dental buds of mouse embryos. Furthermore, It had been reported that *P53* was expressed in all layers of the human tooth buds (Muica Nagy-Bota et al., 2014). Recently, (Zheng et al., 2019) found that *MDM2* promoted the odontoblast-like differentiation of mouse dental papilla cells (mDPCs) by ubiquitinating both *DLX3* and *P53*. Here, we confirmed that *MDM2* was the target gene of *miR-605-5p* and found a negative correlation between *MDM2* and *P53* in dental pulp stem cells. Thus, we hypothesized that the *MDM2: miR-605: P53* feedback loop may also exist during tooth development and rs2043556, significantly associated with differential expression of *miR-605*, contributed to the risk of tooth agenesis probably by influencing this loop (Supplementary Figure S3).

Taken together, this is the first study to evaluate SNPs in miRNA and non-syndromic tooth agenesis susceptibility in a relatively large sample size. Our findings indicated that *miR-605* rs2043556 was associated with risk of non-syndromic tooth agenesis probably by affecting *MDM2: miR-605: P53* feedback loop.

## Data Availability Statement

The datasets presented in this study can be found in online repositories. The names of the repository/repositories and accession number(s) can be found in the article/Supplementary Material.

## Ethics Statement

The studies involving human participants were reviewed and approved by Nanjing Medical University Institution Review Board (PJ2004-030-001). Written informed consent to participate in this study was provided by the participants’ legal guardian/next of kin.

## Author Contributions

LW and YP directed the study, obtained the financial support and were responsible for the study design, interpretation of results, and manuscript writing. MG performed the overall project management with LF. XY performed the statistical analyses with SL and FY, and drafted the initial manuscript with GZ. LM directed each participating study and jointly organized the study. FY and SL were responsible for the sample processing and managed the genotyping data. LF and XY were responsible for the subject recruitment and sample collection. All authors approved the final manuscript.

## Conflict of Interest

The authors declare that the research was conducted in the absence of any commercial or financial relationships that could be construed as a potential conflict of interest.

## Footnotes

1 http://microrna.sanger.ac.uk/

2 http://ncbi.nlm.nih.gov/SNP

3 http://www.hapmap.org

4 http://www.patrocles.org/

5 http://www.ncbi.nlm.nih.gov/geo
