# Supplementary material for: Genetic Variants in miRNAs Are Associated With Risk of Non-syndromic Tooth Agenesis
Source: Front Physiol. 2020 Aug 21;11:1052. doi: 10.3389/fphys.2020.01052 (PMC7472694; doi:10.3389/fphys.2020.01052)

**Figure S1.** The *MDM2* 3ʹ-UTR fragment (407bp) was inserted at the Nhel-Xhol site downstream of the luciferase gene in the pmirGLO vector.
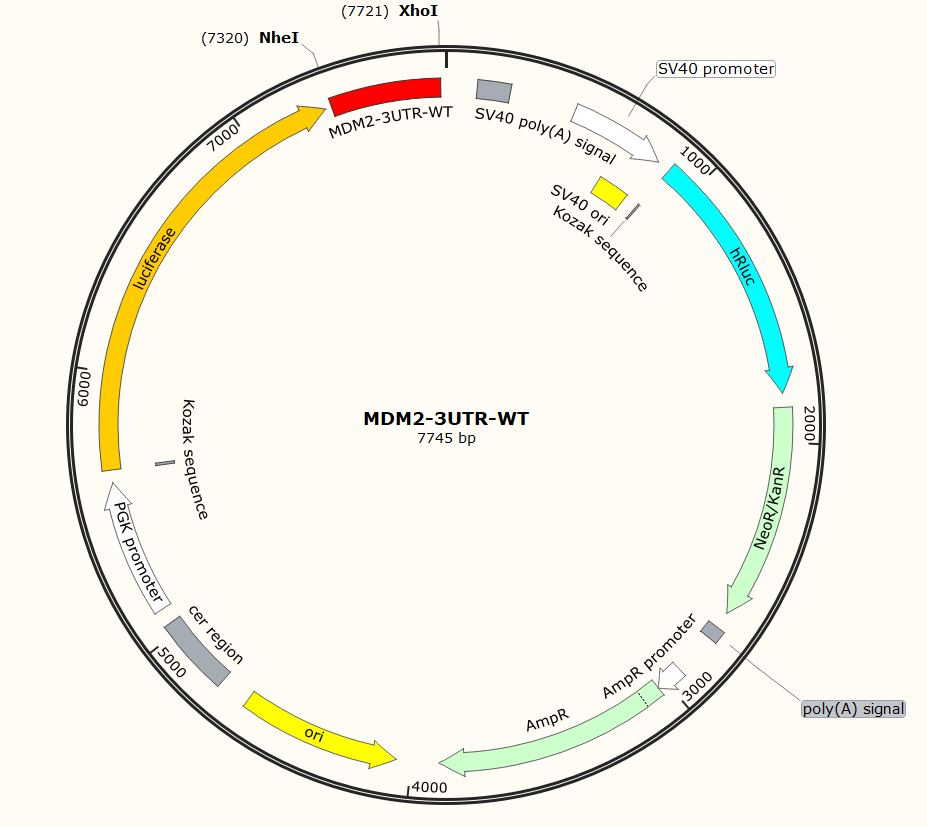

Supplement: Supplementary file 1 [file Table_1.DOCX]
