# Supplementary material for: Genetic Variants in miRNAs Are Associated With Risk of Non-syndromic Tooth Agenesis
Source: Front Physiol. 2020 Aug 21;11:1052. doi: 10.3389/fphys.2020.01052 (PMC7472694; doi:10.3389/fphys.2020.01052)
Supplement: Supplementary file 2 [file Table_2.DOC]

*MDM2* 3' UTR 5’ … AAAAAAAUCCUUUAUGGGAUUUA 3’

[*miR-605-5p*](http://www.mirbase.org/cgi-bin/mirna_entry.pl?acc=hsa-miR-605-5p) 3’   UCCUCUUCCGUGGUACCCUAAAU... 5’

**Figure S2**. The putative *miR-605-5p* binding sequence in the *MDM2* untranslated region (UTR).
