# Supplementary material for: Genetic Variants in miRNAs Are Associated With Risk of Non-syndromic Tooth Agenesis
Source: Front Physiol. 2020 Aug 21;11:1052. doi: 10.3389/fphys.2020.01052 (PMC7472694; doi:10.3389/fphys.2020.01052)
Supplement: Supplementary file 3 [file Table_3.DOC]

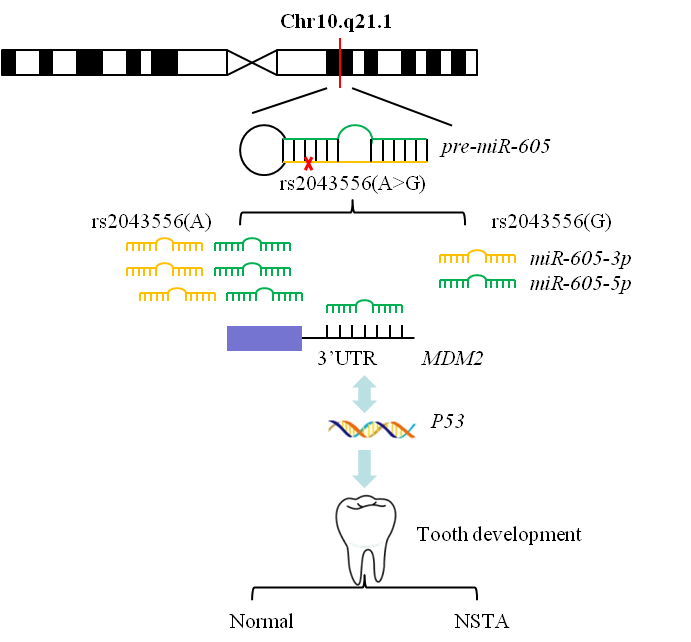


**Figure S3.** **Schematic diagram of the present study**. *Pre-miR-605*/rs2043556 was associated with risk of NSTA probably by affecting *MDM2: miR-605: P53* feedback loop.
