# Supplementary material for: Genetic Variants in miRNAs Are Associated With Risk of Non-syndromic Tooth Agenesis
Source: Front Physiol. 2020 Aug 21;11:1052. doi: 10.3389/fphys.2020.01052 (PMC7472694; doi:10.3389/fphys.2020.01052)
Supplement: Supplementary file 4 [file Table_4.DOC]

| **Characteristics** | **Controls** |  | **Cases** | ***P*** |
| --- | --- | --- | --- | --- |
|  | N = 1,144 (%) |  | N = 625 (%) |  |
| **Gender** |  |  |  |  |
| Male | 415 (36.3%) |  | 205 (32.8%) |  |
| Female | 729 (63.7%) |  | 420 (67.2%) | 0.158^a^ |
| **Age** |  |  |  |  |
| (Mean± SD) | 15.88±7.48 |  | 15.91±8.34 | 0.143^b^ |
| **Maxillary teeth agenesis** | - |  |  |  |
| Subtotal | - |  | 219（30.4%） |  |
| Lateral incisor | - |  | 96 (13.3%) |  |
| Canine | - |  | 51 (7.1%) |  |
| Premolar | - |  | 72 (10.0%) |  |
| **Mandibular teeth agenesis** | - |  |  |  |
| Subtotal |  |  | 501（69.6%） |  |
| Incisor | - |  | 362 (50.3%) |  |
| Premolar | - |  | 139 (19.3%) |  |

**Table S1. Characteristics of the study samples**

^a^Chi-Square Test; ^b^Independent-Sample T Test.
