# Supplementary material for: Genetic Variants in miRNAs Are Associated With Risk of Non-syndromic Tooth Agenesis
Source: Front Physiol. 2020 Aug 21;11:1052. doi: 10.3389/fphys.2020.01052 (PMC7472694; doi:10.3389/fphys.2020.01052)
Supplement: Supplementary file 5 [file Table_5.DOC]

| miRNAs | SNPs | Base position | Alleles^a^ | MAF | HWE^b^ |
| --- | --- | --- | --- | --- | --- |
| *miR-146a* | rs2910164 | 160485411 | G/C | 0.279 (C) | 0.0184 |
| *miR-196a2* | rs11614913 | 53991815 | C/T | 0.333 (T) | 0.2775 |
| *pre-miR-605* | rs2043556 | 51299646 | A/G | 0.260 (G) | 0.8175 |
| *pre-miR-618* | rs2682818 | 80935757 | C/A | 0.242 (A) | 0.9373 |

**Table S2. Details of SNPs in this study**

^a^Reference allele listed first; ^b^*P* value of HWE (Hardy–Weinberg Equilibrium);

MAF: minor allele frequency
