# Supplementary material for: Genetic Variants in miRNAs Are Associated With Risk of Non-syndromic Tooth Agenesis
Source: Front Physiol. 2020 Aug 21;11:1052. doi: 10.3389/fphys.2020.01052 (PMC7472694; doi:10.3389/fphys.2020.01052)
Supplement: Supplementary file 6 [file Table_6.DOC]

**Table S3. Sequence of the TaqMan probes and primers**

| SNPs | Probes (5’-3’-MGB) | Primers (5’-3’) |
| --- | --- | --- |
| rs2910164 | FAM-TCAGACCTGTGAAATT | F: GAACTGAATTCCATGGGTTGTGT |
|  | HEX-TCAGACCTCTGAAATT | R: GCCCACGATGACAGAGATATCC |
| rs2043556 | FAM-CCTAACTTGATTCTAAATC | F: CTGTCAGCCTGTAACATAGGTAACCT |
|  | HEX-CAGTCCTAACTTGGTTC | R: TGGGAAAAACAGAGAAGGCACTA |
| rs11614913 | FAM-TAACTCAGACAGTTTCT | F: CCTCGACGAAAACCGACTGA |
|  | HEX-TAACTCAGGCAGTTTC | R: AGGTAGTTTCATGTTGTTGGGATTG |
| rs2682818 | FAM-AGGGTAACCCTGCTT | F: TTATAGATTTTCCATGAGCTGCTGAT |
|  | HEX-CAGGGTAAACCTGCTT | R: GCCAAACTCTACTTGTCCTTCTGAGT |

F: Forward primer; R: Reversed primer
