# Supplementary material for: Genetic Variants in miRNAs Are Associated With Risk of Non-syndromic Tooth Agenesis
Source: Front Physiol. 2020 Aug 21;11:1052. doi: 10.3389/fphys.2020.01052 (PMC7472694; doi:10.3389/fphys.2020.01052)
Supplement: Supplementary file 7 [file Table_7.DOC]

| miRNA | DPSCs_control | | |  | DPSCs_odontogenic differentiation | | | *P*^a^ |
| --- | --- | --- | --- | --- | --- | --- | --- | --- |
|  | Sample1 | Sample2 | Sample3 |  | Sample4 | Sample5 | Sample6 |  |
| *miR-605-3p* | -3.11 | -3.14 | -3.15 |  | -2.67 | -2.80 | -3.32 | 3.67E-01 |
| *miR-605-5p* | 1.67 | 1.66 | 1.50 |  | 2.61 | 2.44 | 2.81 | **1.12E-03** |
| *P*^b^ | **1.27E-07** | | |  | **1.62E-05** | | |  |

**Table S4**. *miR-605-3p* and *miR-605-5p* expressions in human dental pulp stem cells (DPSCs)

^a^*P* value of differentiated groups comparsion with control groups;

^b^*P* value of *miR-605-3p* expression comparsion with *miR-605-5p* expression;

Bold values: significant values
