# Supplementary material for: Genetic Variants in miRNAs Are Associated With Risk of Non-syndromic Tooth Agenesis
Source: Front Physiol. 2020 Aug 21;11:1052. doi: 10.3389/fphys.2020.01052 (PMC7472694; doi:10.3389/fphys.2020.01052)
Supplement: Supplementary file 8 [file Table_8.DOCX]

| Gene | Transcript _ID | Canine | | | |  | Molar | | | |  | *P*^a^ | Incisor | | | | *P*^b^ |
| --- | --- | --- | --- | --- | --- | --- | --- | --- | --- | --- | --- | --- | --- | --- | --- | --- | --- |
|  |  | sample1 | sample2 | sample3 | average |  | sample1 | sample2 | sample3 | average |  |  | sample1 | sample2 | sample3 | average |  |
| TP53 | 201746_at | 7.54 | 6.36 | 5.29 | 6.40 |  | 6.67 | 6.01 | 5.39 | 6.02 |  | 0.09 | 6.84 | 5.90 | 5.10 | 5.94 | **0.04** |
|  | 211300_s_at | 5.99 | 4.53 | 4.40 | 4.98 |  | 4.56 | 5.13 | 3.72 | 4.47 |  |  | 5.65 | 3.98 | 3.75 | 4.46 |  |
|  | total | 13.54 | 10.89 | 9.69 | 11.38 |  | 11.23 | 11.14 | 9.10 | 10.49 |  |  | 12.49 | 9.89 | 8.85 | 10.40 |  |
|  |  |  |  |  |  |  |  |  |  |  |  |  |  |  |  |  |  |
| MDM2 | 205385_at | 3.75 | 4.11 | 2.09 | 3.32 |  | 3.99 | 4.16 | 2.26 | 3.47 |  | 0.63 | 3.71 | 4.03 | 2.42 | 3.39 | 0.23 |
|  | 205386_s_at | 3.56 | 3.33 | 1.84 | 2.91 |  | 2.86 | 3.40 | 1.95 | 2.74 |  |  | 3.24 | 2.98 | 1.54 | 2.58 |  |
|  | 211832_s_at | 3.34 | 4.22 | 2.36 | 3.31 |  | 3.78 | 3.89 | 2.37 | 3.35 |  |  | 3.34 | 3.58 | 2.10 | 3.01 |  |
|  | 217373_x_at | 4.59 | 3.84 | 2.86 | 3.76 |  | 3.61 | 4.03 | 2.05 | 3.23 |  |  | 4.66 | 4.12 | 3.11 | 3.96 |  |
|  | 217542_at | 4.48 | 6.64 | 4.03 | 5.05 |  | 5.51 | 6.67 | 3.72 | 5.30 |  |  | 3.83 | 7.18 | 4.11 | 5.04 |  |
|  | 225160_x_at | 7.65 | 7.04 | 6.54 | 7.08 |  | 6.77 | 7.74 | 6.37 | 6.96 |  |  | 7.68 | 7.38 | 6.33 | 7.13 |  |
|  | 229711_s_at | 7.38 | 9.38 | 7.68 | 8.15 |  | 8.15 | 9.44 | 7.72 | 8.44 |  |  | 7.62 | 9.50 | 7.75 | 8.29 |  |
|  | 237891_at | 3.20 | 3.81 | 2.00 | 3.00 |  | 3.19 | 3.68 | 2.05 | 2.98 |  |  | 3.24 | 2.95 | 2.00 | 2.73 |  |
|  | 238733_at | 3.53 | 4.50 | 2.55 | 3.52 |  | 3.45 | 4.38 | 2.48 | 3.44 |  |  | 3.52 | 4.08 | 2.42 | 3.34 |  |
|  | 244616_x_at | 6.88 | 7.08 | 5.56 | 6.50 |  | 6.06 | 7.35 | 5.52 | 6.31 |  |  | 6.25 | 7.19 | 5.59 | 6.34 |  |
|  | total | 48.35 | 53.95 | 37.50 | 46.60 |  | 47.37 | 54.74 | 36.50 | 46.22 |  |  | 47.09 | 52.99 | 37.35 | 45.81 |  |

**Table S5. The expression of *p53* and *MDM2* in the human embryonic tooth germs**

^a^*P* value of canine germs comparsion with molar germs by the paired *t* test;

^b^*P* value of canine germs comparsion with incisor germs by the paired *t* test;

Bold values: significant values
